# Supplementary material for: Modeling glioblastoma heterogeneity as a dynamic network of cell states
Source: Mol Syst Biol. 2021 Sep 16;17(9):e10105. doi: 10.15252/msb.202010105 (PMC8444284; doi:10.15252/msb.202010105)
Supplement: Supplementary file 5 — Source Data for Figure 3 [file MSB-17-e10105-s001.zip › Figure3A_sourcedata/GSEA_3065/nosigdb_state2.GseaPreranked.1622718419215/GAROFANO_IAVARONE_LIST_OF_NEURAL_PROGENITOR_MARKER_GENES.html]

Details for gene set GAROFANO\_IAVARONE\_LIST\_OF\_NEURAL\_PROGENITOR\_MARKER\_GENES[GSEA]

|  || Dataset | state2 |
| Phenotype | NoPhenotypeAvailable |
| Upregulated in class | na\_pos |
| GeneSet | GAROFANO\_IAVARONE\_LIST\_OF\_NEURAL\_PROGENITOR\_MARKER\_GENES |
| Enrichment Score (ES) | 0.76827073 |
| Normalized Enrichment Score (NES) | 2.5340445 |
| Nominal p-value | 0.0 |
| FDR q-value | 0.0 |
| FWER p-Value | 0.0 |
Table: GSEA Results Summary

  

Fig 1: Enrichment plot: GAROFANO\_IAVARONE\_LIST\_OF\_NEURAL\_PROGENITOR\_MARKER\_GENES      
 Profile of the Running ES Score & Positions of GeneSet Members on the Rank Ordered List

  

| PROBE | GENE SYMBOL | GENE\_TITLE | RANK IN GENE LIST | RANK METRIC SCORE | RUNNING ES | CORE ENRICHMENT || 1 | HMGB2 |  |  | 4 | 0.823 | 0.0388 | Yes |
| 2 | UBE2C |  |  | 9 | 0.704 | 0.0720 | Yes |
| 3 | DHFR |  |  | 11 | 0.644 | 0.1026 | Yes |
| 4 | RRM2 |  |  | 22 | 0.556 | 0.1281 | Yes |
| 5 | CKS1B |  |  | 23 | 0.554 | 0.1545 | Yes |
| 6 | CKS2 |  |  | 28 | 0.532 | 0.1795 | Yes |
| 7 | TPX2 |  |  | 29 | 0.531 | 0.2049 | Yes |
| 8 | ESCO2 |  |  | 30 | 0.530 | 0.2301 | Yes |
| 9 | DEK |  |  | 31 | 0.526 | 0.2552 | Yes |
| 10 | HMGN2 |  |  | 35 | 0.512 | 0.2794 | Yes |
| 11 | TMPO |  |  | 43 | 0.489 | 0.3019 | Yes |
| 12 | PBK |  |  | 46 | 0.484 | 0.3248 | Yes |
| 13 | ANP32B |  |  | 48 | 0.474 | 0.3473 | Yes |
| 14 | KPNA2 |  |  | 50 | 0.472 | 0.3697 | Yes |
| 15 | TUBA1B |  |  | 52 | 0.462 | 0.3917 | Yes |
| 16 | UHRF1 |  |  | 57 | 0.454 | 0.4129 | Yes |
| 17 | FBXO5 |  |  | 67 | 0.420 | 0.4320 | Yes |
| 18 | EZH2 |  |  | 70 | 0.417 | 0.4517 | Yes |
| 19 | RRM1 |  |  | 72 | 0.412 | 0.4712 | Yes |
| 20 | NUCKS1 |  |  | 78 | 0.404 | 0.4899 | Yes |
| 21 | H2AFV |  |  | 82 | 0.397 | 0.5085 | Yes |
| 22 | DNAJC9 |  |  | 103 | 0.373 | 0.5242 | Yes |
| 23 | ANLN |  |  | 111 | 0.358 | 0.5405 | Yes |
| 24 | SLBP |  |  | 112 | 0.357 | 0.5576 | Yes |
| 25 | TUBB4B |  |  | 125 | 0.340 | 0.5725 | Yes |
| 26 | ANP32E |  |  | 136 | 0.330 | 0.5872 | Yes |
| 27 | ARHGAP11A |  |  | 145 | 0.321 | 0.6016 | Yes |
| 28 | RAD21 |  |  | 147 | 0.316 | 0.6166 | Yes |
| 29 | CKAP2 |  |  | 151 | 0.308 | 0.6310 | Yes |
| 30 | NASP |  |  | 156 | 0.306 | 0.6451 | Yes |
| 31 | PAICS |  |  | 187 | 0.291 | 0.6558 | Yes |
| 32 | NUF2 |  |  | 188 | 0.287 | 0.6695 | Yes |
| 33 | SUZ12 |  |  | 239 | 0.258 | 0.6765 | Yes |
| 34 | DBI |  |  | 251 | 0.252 | 0.6873 | Yes |
| 35 | MIS18BP1 |  |  | 257 | 0.246 | 0.6986 | Yes |
| 36 | LIG1 |  |  | 259 | 0.246 | 0.7102 | Yes |
| 37 | SAE1 |  |  | 278 | 0.242 | 0.7198 | Yes |
| 38 | TCF19 |  |  | 318 | 0.226 | 0.7264 | Yes |
| 39 | CKAP2L |  |  | 327 | 0.224 | 0.7363 | Yes |
| 40 | RACGAP1 |  |  | 333 | 0.222 | 0.7463 | Yes |
| 41 | NUDC |  |  | 346 | 0.218 | 0.7555 | Yes |
| 42 | RPA2 |  |  | 367 | 0.212 | 0.7635 | Yes |
| 43 | SPAG5 |  |  | 439 | 0.192 | 0.7650 | Yes |
| 44 | PRC1 |  |  | 514 | 0.178 | 0.7656 | Yes |
| 45 | OXCT1 |  |  | 624 | 0.160 | 0.7616 | Yes |
| 46 | KIF2A |  |  | 633 | 0.158 | 0.7683 | Yes |
| 47 | PMF1 |  |  | 800 | 0.138 | 0.7571 | No |
| 48 | FABP7 |  |  | 810 | 0.137 | 0.7627 | No |
| 49 | IDI1 |  |  | 878 | 0.130 | 0.7617 | No |
| 50 | PTBP1 |  |  | 943 | 0.124 | 0.7608 | No |
| 51 | AHCY |  |  | 959 | 0.123 | 0.7651 | No |
| 52 | SOX2 |  |  | 1036 | 0.117 | 0.7625 | No |
| 53 | RPL22L1 |  |  | 1112 | 0.111 | 0.7598 | No |
| 54 | RANGAP1 |  |  | 1157 | 0.107 | 0.7602 | No |
| 55 | COX7C |  |  | 1181 | 0.106 | 0.7628 | No |
| 56 | TCF12 |  |  | 1267 | 0.101 | 0.7585 | No |
| 57 | HES6 |  |  | 1305 | 0.098 | 0.7592 | No |
| 58 | SOX9 |  |  | 1466 | 0.088 | 0.7463 | No |
| 59 | YBX3 |  |  | 1677 | 0.078 | 0.7276 | No |
| 60 | ASCL1 |  |  | 2318 | 0.053 | 0.6617 | No |
| 61 | SALL1 |  |  | 2372 | 0.051 | 0.6585 | No |
| 62 | MT1M |  |  | 2562 | 0.046 | 0.6405 | No |
| 63 | BMI1 |  |  | 2913 | 0.036 | 0.6048 | No |
| 64 | EFHD2 |  |  | 3081 | 0.032 | 0.5884 | No |
| 65 | PAX6 |  |  | 3226 | 0.029 | 0.5744 | No |
| 66 | ZIC1 |  |  | 3668 | 0.021 | 0.5283 | No |
| 67 | GLI2 |  |  | 3744 | 0.020 | 0.5212 | No |
| 68 | TP53I11 |  |  | 3751 | 0.020 | 0.5215 | No |
| 69 | NDE1 |  |  | 3764 | 0.019 | 0.5211 | No |
| 70 | INSM1 |  |  | 3801 | 0.019 | 0.5182 | No |
| 71 | MSI1 |  |  | 4226 | 0.012 | 0.4734 | No |
| 72 | CLIC4 |  |  | 4778 | 0.003 | 0.4147 | No |
| 73 | HES1 |  |  | 6191 | -0.020 | 0.2646 | No |
| 74 | NES |  |  | 6455 | -0.025 | 0.2377 | No |
| 75 | MYO10 |  |  | 6758 | -0.031 | 0.2069 | No |
| 76 | FGFR2 |  |  | 6969 | -0.036 | 0.1861 | No |
| 77 | NBL1 |  |  | 7312 | -0.045 | 0.1517 | No |
| 78 | POU3F2 |  |  | 7454 | -0.049 | 0.1390 | No |
| 79 | SFRP1 |  |  | 8103 | -0.071 | 0.0731 | No |
| 80 | NOTCH2 |  |  | 8358 | -0.086 | 0.0500 | No |
| 81 | DLL3 |  |  | 8640 | -0.106 | 0.0251 | No |
| 82 | VIM |  |  | 8691 | -0.110 | 0.0250 | No |
| 83 | NOTCH1 |  |  | 8763 | -0.118 | 0.0230 | No |
| 84 | WNT7B |  |  | 8822 | -0.125 | 0.0228 | No |
| 85 | DLG1 |  |  | 8857 | -0.130 | 0.0254 | No |
| 86 | CALU |  |  | 9278 | -0.223 | -0.0089 | No |
| 87 | CLU |  |  | 9430 | -0.547 | 0.0011 | No |
Table: GSEA details [plain text format]

  

Fig 2: GAROFANO\_IAVARONE\_LIST\_OF\_NEURAL\_PROGENITOR\_MARKER\_GENES: Random ES distribution      
 Gene set null distribution of ES for **GAROFANO\_IAVARONE\_LIST\_OF\_NEURAL\_PROGENITOR\_MARKER\_GENES**
